# Meiotic crossovers characterized by haplotype-specific chromosome painting in maize

**DOI:** 10.1038/s41467-019-12646-z

**Published:** 2019-10-10

**Authors:** Lívia do Vale Martins, Fan Yu, Hainan Zhao, Tesia Dennison, Nick Lauter, Haiyan Wang, Zuhu Deng, Addie Thompson, Kassandra Semrau, Jean-Marie Rouillard, James A. Birchler, Jiming Jiang

**Affiliations:** 1 Department of Plant Biology, Michigan State University, East Lansing, MI 48824 USA; 2 Department of Horticulture, Michigan State University, East Lansing, MI 48824 USA; 3 National Engineering Research Center for Sugarcane, Fujian Agriculture and Forestry University, Fuzhou, China; 4 Genetics and Genomics Graduate Program, Iowa State University, Ames, IA 50011 USA; 5 USDA-ARS Corn Insects and Crop Genetics Research Unit, Iowa State University, Ames, IA 50011 USA; 6 Department of Plant, Soil and Microbial Sciences, Michigan State University, East Lansing, MI 48824 USA; 7 Michigan State University AgBioResearch, East Lansing, MI 48824 USA; 8 Arbor Biosciences, Ann Arbor, MI 48103 USA; 9 Department of Chemical Engineering, University of Michigan, Ann Arbor, MI 48109 USA; 10 Division of Biological Sciences, University of Missouri, Columbia, MO 65211 USA; 11 Present Address: Department of Natural Sciences, University of Michigan-Dearborn, Dearborn, MI 48128 USA

**Keywords:** Cytogenetics, Fluorescence imaging, Cytogenetics, Agricultural genetics

## Abstract

Meiotic crossovers (COs) play a critical role in generating genetic variation and maintaining faithful segregation of homologous chromosomes during meiosis. We develop a haplotype-specific fluorescence in situ hybridization (FISH) technique that allows visualization of COs directly on metaphase chromosomes. Oligonucleotides (oligos) specific to chromosome 10 of maize inbreds B73 and Mo17, respectively, are synthesized and labeled as FISH probes. The parental and recombinant chromosome 10 in B73 x Mo17 F_1_ hybrids and F_2_ progenies can be unambiguously identified by haplotype-specific FISH. Analysis of 58 F_2_ plants reveals lack of COs in the entire proximal half of chromosome 10. However, we detect COs located in regions very close to the centromere in recombinant inbred lines from an intermated B73 x Mo17 population, suggesting effective accumulation of COs in recombination-suppressed chromosomal regions through intermating and the potential to generate favorable allelic combinations of genes residing in these regions.

## Abstract (web summary)

Meiotic crossovers (COs) are essential for proper chromosome segregation and generating novel combinations of alleles. Here, the authors develop haplotype-specific oligos on maize chromosome 10 for fluorescence in situ hybridization and analyze CO patterns in an intermated recombinant population derived from B73 and Mo17.

## Introduction

Meiotic recombination, which generates genetic variation via exchange of DNA between homologous parental chromosomes, is essential for plant and animal breeding. Breeders rely on meiotic recombination to create new and favorable combinations of parental alleles. Meiotic recombination is initiated from the formation of double-strand breaks (DSBs) of DNA molecules^1^. DSBs are then repaired through the double Holliday junction or synthesis-dependent strand annealing pathways, which result in meiotic crossovers (COs) or non-crossovers (NCOs), respectively^2^. COs, which create a physical connection of the homologous chromosome pairs, are also essential for faithful chromosome segregation in meiosis. Thus, essentially every chromosome at the first metaphase of meiosis acquires at least one CO, which is known as the obligatory CO rule^3,4^. The frequency of zero-CO chromosomes is extremely low and is usually much less than 1% in different organisms^4,5^.

Meiotic COs can be mapped on chromosomes using different methods. COs can be indicated based on the locations of late recombination nodules (RNs) or protein markers associated with COs, such as MLH1, on synaptonemal complexes (SCs)^6,7^. However, it is often difficult or impossible to distinguish individual SCs in the same plant species, which would prevent to map each RN on a specific chromosome. Physical mapping of genetically anchored DNA markers can be used to predict the locations of COs on chromosomes. Physical mapping in plants can be achieved by using cytogenetic stocks^8,9^, or by fluorescence in situ hybridization (FISH) of DNA markers directly on chromosomes^10–14^. However, physical mapping can unveil the relationship between genetical and chromosomal distances of the DNA markers, but does not reveal the exact positions of individual COs. Lastly, genomic in situ hybridization (GISH) can be used to visualize COs derived from homoeologous chromosomes^15–17^. GISH, however, relies on presence of dispersed repeats specific to each chromosone, thus, it can not be used to detect COs from homologous chromosomes.

We develop a FISH technique that allows us to visualize meiotic COs derived from homologous plant chromosomes. We computationally identify large sets of oligonucleotides (oligos) that are specific to chromosome 10 of either maize inbred B73 or Mo17, two highly utilized inbreds that belong to two important opposing heterotic groups^18^ and have been used extensively as parental lines for genetic mapping and heterosis studies^19–21^. The identified oligos are based on presence-absence variation (PAV), single nucleotide polymorphisms (SNPs), and/or insertions and deletions (indels) in chromosome 10 sequences derived from the two inbreds. These oligos are then massively synthesized and labeled as FISH probes. We are able to differentially paint the two copies of parental chromosome 10 in a B73 × Mo17 hybrid using these haplotype-specific FISH probes. We intend to use this FISH technique to detect and quantify meiotic COs derived from the two homologous chromosome 10 in different maize populations.

## Results

### Identification of haplotype-specific oligos

Chromosome 10 of maize consists of 150.98 megabases (Mb) of DNA sequences^22,23^. To develop oligo-FISH probes that can be used differentially paint the chromosome 10 from maize inbreds B73 and Mo17, respectively, we used Chorus (https://github.com/forrestzhang/Chorus) to generate single copy oligos (45 nucleotides (nt)) from the pseudomolecules of chromosome 10 of B73^24^ and Mo17^23^. We obtained a total of 175,437 and 174,728 oligos for B73 (B73 oligos) and Mo17 (Mo17 oligos), respectively. We then identified B73 oligos that are absent in the Mo17 genome and vice versa (see Methods section), which were named PAV oligos. B73 oligos that contain mismatches and/or indels to the Mo17 chromosome 10 sequence, and vice versa, were defined as SNP oligos. We only retained oligos with mismatches and/or indels located between 10–35 bp within each oligo. SNP oligos were identified in pairs (see Methods section), one for B73 and one for Mo17. We calculated the ΔTm between the B73 and Mo17 oligos of each pair using primer3^25,26^. Oligo pairs with ΔTm > 5 °C were discarded to avoid hybridization bias towards one variant.

### Chromosome painting using haplotype-specific oligo probes

**Fig. 1:**
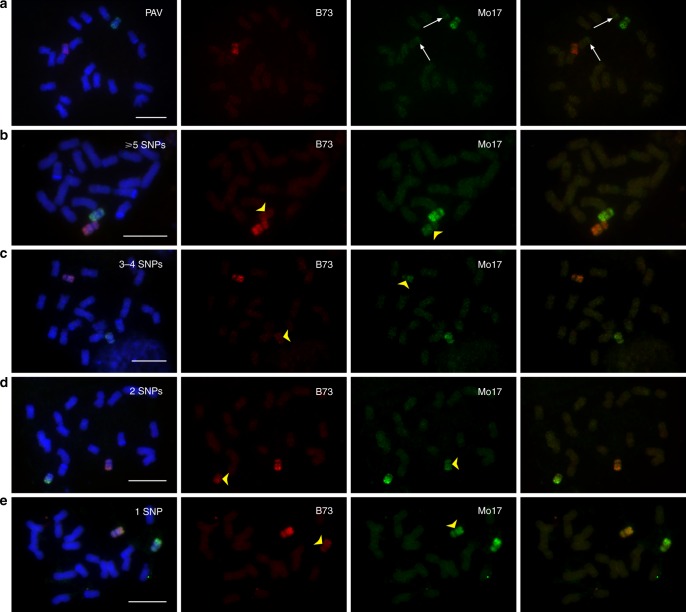
Development of maize chromosome 10 oligo-FISH probes specific to inbreds B73 or Mo17. **a** Two oligo-FISH probes based on presence-absence variation. **b** Two oligo-FISH probes based on 5 or more single nucleotide polymorphisms (SNPs). **c** Two oligo-FISH probes based on 3 or 4 SNPs. **d** Two oligo-FISH probes based on 2 SNPs. **e** Two oligo-FISH probes based on 1 SNP. Oligo-FISH probes specific to the B73 haplotype were detected in red color. Oligo-FISH probes specific to the Mo17 haplotype were detected in green color. Images in first column: Complete metaphase cells hybridized with the two FISH probes; Images in the second column: digitally separated red FISH signals derived from the B73-specific probes; Images in the third column: digitally separated green FISH signals derived from the Mo17-specific probes; Images in the forth column: merged FISH signals derived from both B73 and Mo17. Arrows indicate the cross-hybridization signals at the 45 rDNA region associated with chromosome 6. Yellow arrowheads indicate the cross-hybridization signals from B73-specific probes to Mo17 chromosome 10, and vice versa. Bars = 10 μm. The original gray-scale images used to generate the five color images in the first column of all panels of Fig. 1 are provided as a Source Data file

We identified 6251 and 5506 PAV oligos specific to B73 and Mo17, respectively. These two sets of oligos were synthesized as two oligo pools. The two synthesized pools of oligos were then amplified, labeled, and hybridized to the metaphase chromosomes prepared from a B73 × Mo17 hybrid plant. Each pool of oligos generated distinct FISH signals highly specific to the chromosome 10 derived from B73 (red) or Mo17 (green) (Fig. 1a). The B73 PAV probe generated only minimal cross hybridization to chromosome 10 from Mo17, and vice versa. However, the Mo17 probe hybridized to the 45S ribosomal RNA gene region on chromosome 6 from both inbreds (Fig. 1a). This cross hybridization was found to be caused by three oligos that shared 79–88% sequence similarity with the 45S rRNA genes. These three oligos were not detected by the oligo selection software.

**Fig. 2:**
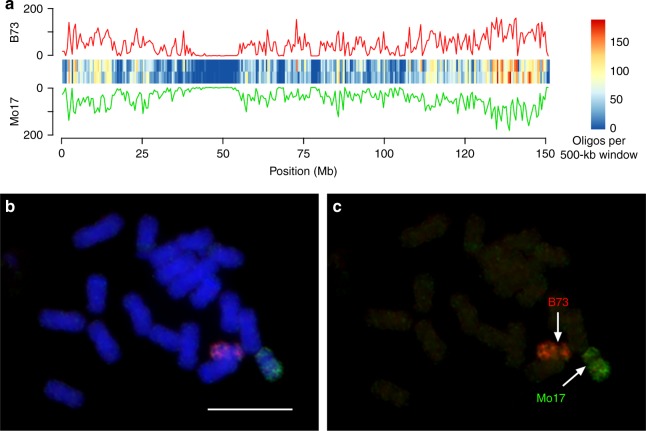
Development of hapB and hapM probes. **a** Distribution of 14,498 oligos (red) of probe hapB and 13,753 oligos (green) of probe hapM on maize chromosome 10. The chromosome was divided into 500-kb windows and number of oligos was calculated for each window. The distribution of the number of oligos is shown in the line plot and heatmap. *y*-axis is the number of oligos in each 500-kb window. **b** Oligo-FISH on metaphase chromosomes from a B73 × Mo17 hybrid using hapB (red) and hapM (green). Bar = 10 μm. **c** The oligo-FISH signals were digitally separated from panel (**b**). The original gray-scale images used to generate the color image in Fig. 2b are provided as a Source Data file

We identified 4353 pairs of oligos that are differentiated by 5 or more SNPs between the B73 and Mo17 chromosome 10 sequences. These two sets of oligos generated strong signals on the respective chromosome 10 from B73 or Mo17. We observed weak but visible cross hybridization of the B73 probe to the Mo17 chromosome, and vice versa (Fig. 1b). Similarly, we identified 3894, 6506, and 19,885 pairs of oligos that are differentiated by 3–4 SNPs, 2 SNPs, and 1 SNP, respectively, between the B73 and Mo17 chromosome 10 sequences. Each SNP probe generated stronger signals on the respective chromosome 10 than on the other copy of chromosome 10 in the hybrid. However, the cross hybridization signals became stronger as the number of SNP decreased (Fig. 1). We then used different combinations of these five pairs of oligo pools. The combined pools of oligos with PAV, ≥5 SNPs, and 3–4 SNPs produced the best contrast of haplotype-specific FISH signals (Fig. 2b, c). These two probe pools, thereafter named hapB (haplotype B73, red) and hapM (haplotype Mo17, green), and were used in all future FISH experiments.

Probes hapB and hapM contain 14,498 and 13,753 oligos, respectively. These oligos are not uniformly distributed on chromosome 10 (Fig. 2a). The chromosomal region spanning 42–54 Mb, which includes the centromere^27^, contained only 27 and 39 oligos in B73 and Mo17, respectively. Similarly, only 74 (115) oligos were identified in the first 2 Mb on the short arm and 19 (0) oligos identified in the final 1 Mb on the long arm of B73 (Mo17) (Supplementary Data 1). Thus, these regions showed weak or undetectable FISH signals (Fig. 2c).

### Meiotic COs revealed by chromosome painting

**Fig. 3:**
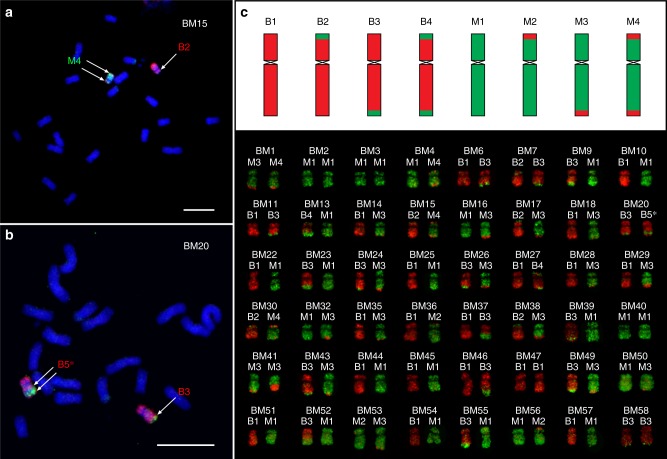
Crossovers between B73 and Mo17 chromosome 10 revealed by oligo-FISH mapping using probes hapB and hapM. **a** Oligo-FISH mapping of B73 × Mo17 F_2_ plant BM15. Probes hapB and hapM are shown in red and green, respectively. The single arrow identifies a single chromosomal exchange position (EP) on the B2-classified chromosome. Double arrows point to the two chromosomal EPs on the M4-classified chromosome. Bar = 10 μm. **b** Oligo-FISH mapping of B73 × Mo17 F_2_ plant BM20. The single arrow points a single EP on the B3-classified chromosome. Double arrows point to the two EPs of double crossovers (COs) on the B5^∗^-classified chromosome. Bar = 10 μm. **c** Upper panel: diagrams of the 8 types of parental or recombinant chromosomes identified in F_2_ plants. B indicates B73; M indicates Mo17. Lower panel, representative images of the two copies of chromosomes 10 in 48 F_2_ plants. One copy of chromosome 10 in BM20 is marked as B5^∗^, which is the only chromosome that does not belong to any of the eight types listed in the upper panel. The original gray-scale images used to generate the color images in Figs. 3a, b are provided as a Source Data file

We wanted to exploit the potential of oligo-FISH using hapB and hapM to detect meiotic COs derived from the homologous chromosome 10 from B73 and Mo17. We produced F_2_ seeds by pollinating sibling B73 × Mo17 hybrid plants. Each F_2_ plant contains two copies of chromosome 10, with each homolog classified as parental or recombinant (Fig. 3). We performed oligo-FISH experiments on somatic metaphase chromosomes from 58 F_2_ plants (BM1-BM58), resulting in the analysis of 116 copies of chromosome 10. These chromosomes can be cataloged as eight different types based on the positions of chromosomal exchanges (Fig. 3c). We identified at least one unambiguous B73-Mo17 chromosomal exchange on 50 (43%) of the 116 chromosomes (Supplementary Data 2), including 6 chromosomes with an exchange on both arms (Fig. 3a). A chromosome 10 with three or more COs was not identified in the analysis.

**Fig. 4:**
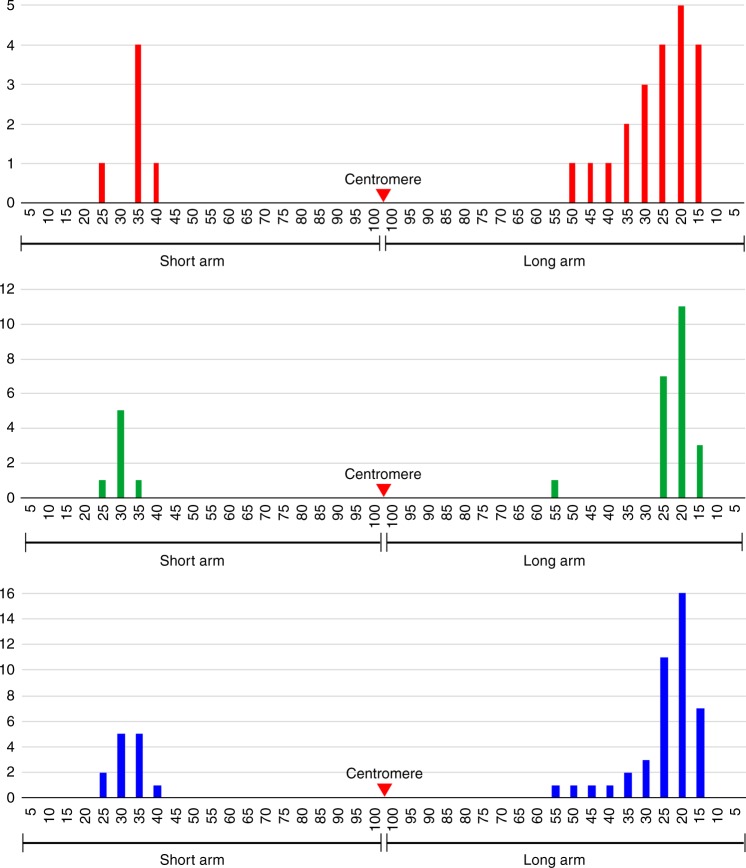
Distribution of chromosomal exchange positions on chromosome 10. Upper panel, middle panel, and lower panel show exchange positions (EPs) on chromosome 10 of B73, Mo17, and combined chromosome 10, respectively. Each interval on the *x*-axes represents 5% of the length of the short or long arm. *y-*axes indicate the number of EPs within a particular 5% of the short arm or long arm. The red triangles point to the centromere position on the chromosome

We measured the distance (μm) from each homologous chromosome exchange position (EP) to the telomere of the respective chromosome arm. This distance was divided by the total length of the respective chromosome arm to calculate the FLA (Fractional Length of the Arm) (see Methods section), which was used to map the position of each EP on the arm (Fig. 4). For example, if the FLA of an EP is 42.5 on the long arm, the distance from this EP to the telomere is 42.5% of the long arm. We divided the short and long arms of the chromosome into 100 intervals, from 0 at the telomere to 100 at the centromere. Each EP was then mapped to one of these intervals (Fig. 4). Most EPs were located within intervals 20–40 on the short arm and intervals 10–55 on the long arm (Fig. 4). Thus, no EP was observed within nearly half of the chromosome 10 flanking the centromere (Fig. 4). In addition, double COs in the same arm were rare and were found only in one of the 116 copies of chromosome 10 analyzed (Fig. 3b).

### Validation of crossovers by genomic sequencing

**Fig. 5:**
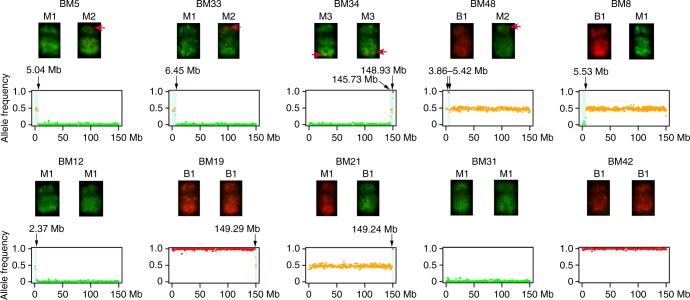
Oligo-FISH and sequencing analyses of 10 F_2_ plants. Red arrows indicate the B73 FISH signals located on Mo17-derived chromosome 10 in BM5, BM33, BM34, and BM48. These crossovers (COs) were inconsistently detected in different metaphase cells in each line. Black arrows mark the chromosomal exchange position on chromosome 10. No CO was detected in any of the chromosome 10 in BM31 and BM42. The *y*-axis of each sequencing profile represents the allele frequency of B73 (red) or Mo17 (green). The *x*-axis represents the sequence position of chromosome 10 (150.98 Mb). Orange dots indicate the mix of red (B73 allele) and green (Mo17 allele) dots

Recombinant or parental copies of chromosome 10 were unambiguously identified in 54 of the 58 F_2_ plants analyzed. In the remaining four F_2_ plants (BM5, BM33, BM34, BM48) (Supplementary Data 2), we observed putative recombinant chromosome(s) with faint FISH signals that were inconsistently observed in the metaphase cells analyzed (Fig. 5). We conducted genomic sequencing of these four plants to validate the inconsistent identification of the COs. In addition, no COs were identified in 18 of 58 (31%) F_2_ plants. We suspected that CO(s) may occur at the distal regions on chromosome 10 in these plants. These COs may not be detectable by FISH due to the lack of a sufficient number of oligos selected from the distal regions (Fig. 2a, Supplementary Data 1). Therefore, we also included six F_2_ plants (BM8, BM12, BM19, BM21, BM31, BM42), which did not have visible COs, in the sequencing analysis. These 10 plants included 13 copies of chromosome 10 without a visible CO. We generated an average of 2.41× coverage of 150 bp paired-end Illumina sequences from each of the 10 plants for the analysis.

BM5 contained two copies of Mo17-derived chromosome 10. A CO was detected on the short arm of one chromosome 10 in 8 of 25 (32%) metaphase cells analyzed (Fig. 5). Sequencing analysis showed that one chromosome 10 has a CO at 5.04 Mb (Fig. 5). Thus, the oligo-FISH result was supported by the sequencing data. Similarly, BM33 also contained two copies of Mo17-derived chromosome 10. FISH detected a CO on the short arm of one chromosome 10 in 11 of 19 (58%) metaphase cells analyzed. Sequencing analysis revealed a CO at 6.45 Mb on the short arm of one chromosome (Fig. 5), thereby supporting the CO observed with oligo-FISH.

BM34 contained two copies of Mo17-derived chromosome 10. We observed hapB FISH signals at the distal regions of the long arms of both chromosome 10 homologs in 6 of 29 (21%) metaphase cells analyzed (Fig. 5). Sequencing analysis revealed a single CO on the long arm of each chromosome 10 homolog: at 145.73 Mb (5.25 Mb away from the end of the long arm telomere) and 148.93 Mb (2.06 Mb away from the long arm telomere), respectively (Fig. 5). Thus, the sequencing data confirmed the locations of COs on the long arms of both chromosomes.

BM48 contained one B73-derived and one Mo17-derived chromosome 10. We observed weak hapB FISH signals on the Mo17-derived chromosome 10, and weak hapM FISH signals on the B73-derived chromosome 10, but only in 35% (9/26) and 8% (2/26) of the metaphase cells, respectively. Sequencing analysis identified a duplicated B73 fragment between 3.86 Mb and 5.42 Mb on the short arm (Fig. 5). This fragment was possibly resulted from a putative double COs occurred at 3.86 Mb and 5.42 Mb, respectively, or two single COs, one at 3.86 Mb on the B73-derived chromosome 10, and one at 5.42 Mb on the Mo17-derived chromosome 10 (Fig. 5).

COs were not detected by oligo-FISH in BM8, BM12, BM19, and BM21. However, a single CO was discovered on one copy of chromosome 10 in each of these four lines by sequence analysis. The EPs of these four COs were 5.53 Mb, 2.37 Mb, 1.69 Mb, and 1.74 Mb away from the end of the chromosome, respectively (Fig. 5). COs were not detected in BM31 and BM42 using oligo-FISH or sequencing analysis (Fig. 5).

In summary, genomic sequencing of 10 F_2_ plants revealed COs in 9 of the 20 copies of chromosome 10, including 4 copies of recombinant chromosome 10 that were FISH negative. No COs were detected in BM31 and BM42 (Fig. 5). The comparative analysis showed that COs cannot be reliably detected by the hapB and hapM probes if the EP is located <5 Mb away from the telomere of a chromosome arm, which contain only ~600 oligos within each probe (Supplementary Data 1).

### Characterization of recombinant inbred lines

B73 and Mo17 have been popular parental lines used in genetic mapping of maize. An intermated B73 × Mo17 recombinant inbred line (IBMRIL) population was developed by randomly intermating plants for four generations following the F_2_ generation^28^. The increased recombination in this population has resulted in a nearly 4-fold increase in the genetic map distance compared with conventional nonintermated RIL populations^29^. Thus, chromosomes in IBMRIL are expected to contain four times of more COs compared to the F_2_ plants from the B73 × Mo17 hybrids. We intended to use oligo-FISH to cytologically characterize the multiple COs on chromosome 10, which were delineated by genotype and genome sequence data^30,31^.

**Fig. 6:**
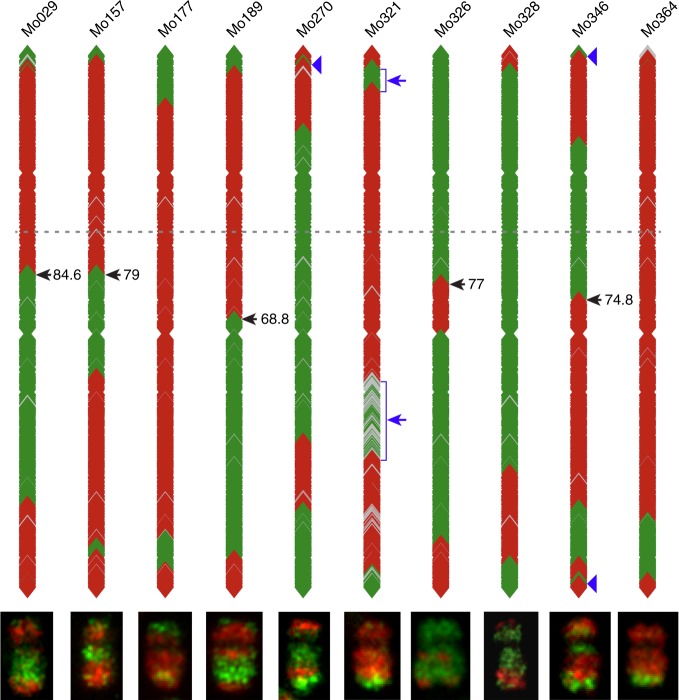
Schematic illustration of genotyping data and oligo-FISH results of 10 lines from the IBMRIL population. The genotyping data is illustrated by location of markers from B73 (red) and Mo17 (green), respectively. Gray indicates missing genotyping data. A dashed line marks the putative position of the centromere on each of the marker-based diagrams. The regions indicated by blue arrows on Mo321 were not consistently visualized by oligo-FISH. Blue arrowheads on Mo270 and Mo346 point to small regions that were not identified by oligo-FISH. Black arrows point to the chromosomal exchange positions (EPs) and their fractional length of the arm (FLA), all on the long arm

**Fig. 7:**
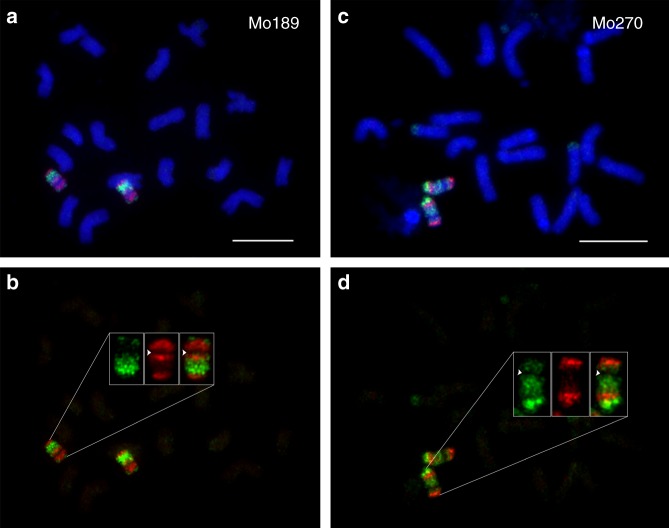
Oligo-FISH characterization of two lines from the IBMRIL population. **a** Oligo-FISH analysis of Mo189. Bar = 10 μm. **b** The oligo-FISH signals of the two chromosome 10 were digitally separated from panel (**a**). Signals from one copy of chromosome 10 are exemplified. Two B73 chromosomal blocks (red), including the centromere, and two Mo17 blocks (green) are identified on chromosome 10. Arrowheads indicate the centromeric region that shows weak signals due to few oligos available. **c** Oligo-FISH mapping of Mo270. Bar = 10 μm. **d** The oligo-FISH signals of the two chromosome 10 were digitally separated from panel (**c**). Signals from one copy of chromosome 10 are exemplified. Two Mo17 blocks (green), including the centromere and two B73 blocks (red) are identified on chromosome 10. Arrowheads indicate the centromeric region that shows weak signals due to few oligos available. The original gray-scale images used to generate the color images in Fig. 7a and c are provided as a Source Data file

We re-analyzed the chromosome 10 genotyping data of the IBMRIL population (see Methods section) and chose 10 IBMRILs for oligo-FISH analysis. A total of 45 genetically immortalized CO events were identified across the 10 IBMRILs using genotypic data sets^30,31^ (Supplementary Table 1). The genotypic data indicated that these 10 IBMRILs contain multiple COs located throughout chromosome 10 (Fig. 6). Several of these IBMRILs contain putative COs located in the pericentromeric regions. FISH using the hapB and hapM probes revealed that nearly all of the recombination events predicted by the genotypic data were associated with a visible CO. For example, line Mo189 was predicted to contain a proximal B73 chromosome segment, including the centromere, and a distal B73 segment on the long arm (Fig. 6, Supplementary Data 3). Oligo-FISH mapping revealed that chromosome 10 of Mo189 contains two chromosomal segments, including the centromere, from B73 and two segments from Mo17 (Figs. 6 and 7a, b). Similarly, both genotyping and oligo-FISH data showed that chromosome 10 of Mo270 contains two segments from Mo17, including the centromere, and two segments from B73 (Figs. 6 and 7c, d). However, we were not able to detect the putative small chromosomal fragments, which were indicated by a single or few DNA markers, such as those associated with lines Mo270 and Mo346 (Fig. 6). These regions may represent small chromosomal segments from B73 or Mo17, but cannot be visualized by oligo-FISH using the hapB and hapM probes.

COs close to the centromere of chromosome 10 were detected in several IBMRILs. For example, an EP on chromosome 10 of Mo189 was mapped at FLA 68.8 on the long arm (Figs. 6 and 7b). The EP that is most close to the centromere was mapped at FLA 84.6 on the long arm of chromosome 10 of Mo029 (Fig. 6). Marker analysis on 209 IBMRILs showed that 11 additional lines (in addition to the five lines in Fig. 6) contain a CO with a breakpoint at the same position as Mo189 or more close to the centromere of chromosome 10. Therefore, the intermating process is highly effective to recover and accumulate the rare CO events occurred in the pericentromeric regions.

## Discussion

COs are not evenly distributed along the chromosomes in most plant species studied so far. Suppression of genetic recombination in the pericentromeric regions has been well documented in a number of plant species^8,9,13^. Physical mapping of a large number of RNs indicated the lack of recombination in the pericentromeric regions of maize chromosomes, although such analysis was conducted only in a single inbred line KYS^32^. The RN-based analysis showed that the pericentromeric regions, which account approximately half of the length of each chromosome, were nearly free of RNs^32^. Recombination suppression in the pericentromeric regions was also supported by physical mapping of genetically achored DNA markers on maize chromosomes^33–35^. We demonstrate that several IBMRILs contain COs close to the centromere of chromosome 10 (Fig. 6), which were not detected in the 58 F_2_ plants (Fig. 4). Thus, the intermating process was effective to accumulate COs in recombination-suppressed chromosomal regions, which can be used to generate favorable allelic combinations of genes residing in these regions.

If a single CO is associated with chromosome 10 during meiosis of the B73 × Mo17 hybrid, two of the four chromatids will be involved in this CO event. Therefore, 50% of the progeny chromosome 10 will become a recombinant chromosome that may be detected by FISH. Our FISH analysis of 58 F_2_ BM plants indicated that 60 of the 116 copies of chromosome 10 are not recombinant chromosomes (Supplementary Data 2). We sequenced 10 F_2_ BM plants, including 13 copies of chromosome 10 that were FISH negative (Fig. 5). Sequence analysis showed that 69% (9/13) of these FISH-negative chromosome 10 were confirmed as non-recombinant chromosomes. Therefore, the percentage of chromosome 10 without CO is estimated to be 36% (69% × 60/116) in the F_2_ population. Luo et al. (2019)^36^ have recently developed a technique to sequence individual gametophyte using a maize hybrid derived from a cross between inbreds Zheng58 and SK. They reported that 18% male gametes and 31% female gametes from this hybrid do not contain a CO on chromosome 10^36^, which would result in 25% of chromosome 10 without CO in F_2_ population. Thus, our data of 36% chromosome 10 without CO in the B73 × Mo17 F_2_ population is higher than the predicted 25% in the Zheng58 × SK F_2_ population. This variation may be caused by the different maize inbreds used in the F_1_ hybrids. In Arabidopsis, meiotic COs on chromosome 4, the smallest chromosome, were analyzed by genotyping F_2_ plants resulting from a cross between ecotypes Columbia and Landsberg^37^. Approximately 30% (423 of 1404) chromosome 4 do not contain a CO^37^, which is close to 36% of non-recombinant maize chromosome 10 in our study.

A major recent advance of FISH is the application of probes based on synthetic oligos^38–40^. Oligo-based chromosome painting probes have been developed in several plant species and applied in various types of chromosomal studies^41–47^. We demonstrate that haplotype-specific oligo-FISH can be to visualize meiotic COs derived from homologous chromosomes derived from different maize inbreds. Although COs can be mapped using RNs or protein markers^7,32^, these traditional methods can only predict the positions of COs on SCs in a specific maize line. In contrast, haplotype-specific FISH maps the chromosomal breakpoints derived from historical COs, including breakpoints derived from multiple COs on recombinant inbred lines from the intermated B73 × Mo17 population (Fig. 7), which can not be analyzed by the traditional methods. The haplotype-specific FISH probes will have unique applications in tracking specific chromosomes derived from a single genotype (Supplementary Note 1). For example, such probes can be used to examine the extent of somatic recombination reported in several plant species^48,49^. Haplotype-specific probes can potentially be used to distinguish true homologous chromosome pairing from pairing of homoeologous chromosome with minor structural variation in polyploid species.

## Methods

### Plant materials and associated genomic data sets

Hybrids between B73 and Mo17 were developed and sibling F_1_ plants were pollinated to generate F_2_ seeds. IBMRIL seed was originally obtained from the Maize Genetics Cooperative Stock Center and was bulked and quality-controlled as described^50^. This ensured correspondence with the genotype data used to construct the original IBM2 map^30^, which are available at MaizeGDB^51^. To add genetic detail for the chromosome 10 investigation, data for 1761 SNPs genotyped for the IBMRILs^31^ was merged with the 64 markers available in the IBM2:chromosome 10 data set. Physical genomic coordinates of the markers corresponding to the Zm00001d genome^24^ were obtained using resources available at MaizeGDB^51^.

### Development of haplotype-specific oligo-FISH probes

Chorus software (https://github.com/forrestzhang/Chorus) was used to generate single copy 45-nt oligos from chromosome 10 of B73^24^ and Mo17^23^, respectively, with a parameter of Chorus “-l 45 –homology 75 –step 5”. B73 reference genome Zm-B73-REFERENCE-GRAMENE-4.0^24^ and Mo17 genome Zm-Mo17-REFERENCE-CAU-1.0^23^ were download from NCBI under project PRJNA10769 and PRJNA358298, respectively. To validate the repetitiveness of each oligo, we generated the frequency distribution of 17-mers from B73 (SRR407544 and SRR407504, JGI) and Mo17 (SRR5826129,^23^) genomic sequencing data. Any 17-mers with a frequency more than 100 in the genome was defined as a repetitive 17-mer. Oligos containing 2 or more repetitive 17-mers were discarded.

In total, 175,437 and 174,728 oligos from B73 and Mo17 chromosome 10 were generated, respectively (Supplementary Note 1). To identify oligos that distinguish the chromosome 10 sequences from the two inbreds, we mapped B73 (Mo17) oligos to Mo17 (B73) reference genome using BWA ALN^52^ with default parameters and blastn in BLAST^53^ with parameter “-task blastn”. B73 (Mo17) oligos that were not identified on Mo17 (B73) chromosome 10 were defined as PAV oligos. B73 (Mo17) oligos containing mismatches and/or indels to Mo17 (B73) chromosome 10 were defined as SNP probe. We only retained SNP oligos with mismatches and/or indels located between 10 and 35 bp within each oligo. For each B73 oligo, the B73 sequences at SNPs and/or indels were replaced by the Mo17 sequences, and vice versa. Therefore, SNP oligos are in pairs, in which one oligo set is homologous to the B73 genome but showed mismatches and/or indels to Mo17 genome and vice versa. We then calculated the ΔTm (difference of melting temperature) between B73 oligo and Mo17 oligo of each oligo pair using primer3^25,26^. We discarded oligo pairs with ΔTm > 5 °C to avoid hybridization bias toward one variant. The SNP oligo pairs were divided into four different classes: oligos with 1 SNP, 2 SNPs, 3–4 SNPs, and 5 or more SNPs. The two probes used for CO characterization, hapB and hapM, included all PAV oligos and oligos with 3–4 SNPs, and 5 or more SNPs. These two probes contained 14,498 and 13,753 oligos, respectively (Supplementary Data 4).

### Oligo-FISH

All seeds were germinated in the laboratory and plants were transferred to the greenhouse. Root tips were collected from the plants and were treated with nitrous oxide at a pressure of 160 psi (~10.9 atm) for 2 h. Root tips were harvested from plants growing in greenhouses. Chromosome preparation from root tips followed published protocols^42^. We synthesized 10 different oligo pools (five for B73, five for Mo17), including two PAV oligo pools, and four paired (i.e., eight total) oligo pools with 1 SNP, 2 SNPs, 3–4 SNPs, or ≥5 SNPs. The B73 oligo probes were labeled with digoxigenin and Mo17 probes were labeled with biotin. Amplification and labeling of the oligo-based probes were according to published protocols^41^. All biotin-labeled probes were detected by anti-biotin fluorescein (Vector Laboratories, Burlingame, California) and digoxigenin-labeled probes were detected by anti-digoxigenin rhodamine (Roche Diagnostics, Indianapolis, Indiana). DAPI (4’,6-diamidino-2-phenylindole) was used to counterstain chromosomes in the VectaShield antifade solution (Vector Laboratories). FISH images were captured using a QImaging Retiga EXi Fast 1394 CCD. The original gray scale images were processed with Meta Imaging Series 7.5 software. The contrast of the gray scale images was adjusted and merged using Adobe Photoshop CS3 software.

For each unambiguously identified meiotic CO we measured the distance, or length (μm), from the chromosomal exchange point (EP) to the telomere of the respective chromosome arm. The length (μm) of the respective chromosome arm bearing the CO was also measured. The chromosomal position of each EP is presented as a FLA (Fractional Length of the Arm) by dividing the measured distance by the total length of the chromosome arm. DRAWID (10.3897/compcytogen.v11i4.20830) was used to measure the distance from the FISH signal to the telomere of the corresponding arm, as well as the length of the entire arm.

### Sequencing and analysis

Genomic DNA was isolated from 10 F_2_ plants (BM5, BM33, BM34, BM48, BM8, BM12, BM19, BM21, BM31, BM42) for Illumina sequencing. We generated an average of 2.41× coverage of 150 bp pair-end sequences from these plants. The sequence data was mapped to the B73 RefGen_v4^24^ by BWA MEM software with default parameters^52^. Reads with mapping quality more than 50 were retained for SNP calling. SNPs were detected using freebayes with parameter “-C 1 –m 50 –q 30”^54^. Raw SNPs were further filtered using vcffilter in freebayes package by “DP < 40” and “QUAL/DP > 10”. The bin map was constructed using a sliding window approach^55^ with minor modification. SNPs were scanned in 300‐SNP‐windows and the genotype of each window defined by the percentage of B73 and Mo17 SNPs: the windows were called homozygous B73 genotype when SNP_B73_ is more than 80% in the window, homozygous Mo17 genotype when SNP_Mo17_ is more than 80% in the window and heterozygous genotype when SNP_B73_ was between 20 and 80%.

### Reporting summary

Further information on research design is available in the Nature Research Reporting Summary linked to this article.

## Supplementary information

Supplementary Information

Peer Review

Reporting Summary

Description of Additional Supplementary Files

Supplementary Data 1

Supplementary Data 2

Supplementary Data 3

Supplementary Data 4

Source Data

## Data Availability

Data supporting the findings of this work are available within the paper and its Supplementary Information files. A reporting summary for this article is available as a Supplementary Information file. The datasets generated and analyzed during the current study are available from the corresponding author upon request. Genomic sequencing data of the 10 maize lines have been deposited to NCBI Sequence Read Archive (SRA) under project PRJNA540894 (https://www.ncbi.nlm.nih.gov/bioproject/?term=PRJNA540894). The source data underlying Figs. 1, 2b, 3a, 3b, 7a, and c are provided as a Source Data file.
