# Supplementary material for: Meiotic crossovers characterized by haplotype-specific chromosome painting in maize
Source: Nat Commun. 2019 Oct 10;10:4604. doi: 10.1038/s41467-019-12646-z (PMC6787048; doi:10.1038/s41467-019-12646-z)
Supplement: Supplementary file 1 — Supplementary Information [file 41467_2019_12646_MOESM1_ESM.pdf]

Meiotic crossovers characterized by haplotype-specific chromosome painting in maize

Martins *et al.*

Supplementary Note 1

Cost of oligo-based FISH probes

Arbor Biosciences (Ann Arbor, Michigan 48103) currently charges $1500 for synthesizing a pool of ~25,000 oligos (45 nt), or three sub-pools with each containing ~6000 oligos. Approximately 200 ng of DNA will be produced for each pool or sub-pool. The DNA is then amplified and labeled as a FISH probe. It was estimated that the 200 ng of synthesized DNA can be used for one million FISH experiments/slides[1]. Thus, each synthesized DNA sample is nearly an infinite probe resource.

Oligo-FISH probes for somatic metaphase chromosomes or meiotic pachytene chromosomes

Although probes containing as few as ~5000 oligos generated detectable FISH signals on maize chromosome 10 prepared from root tip cells (Figure 1), these probes do not generate ideal signals on meiotic pachytene chromosomes. Plant pachytene chromosomes are typically 10-20 times longer than somatic metaphase chromosomes. Thus, a probe with a low density of oligos uniformly distributed to a chromosome will generate significantly more diffused and weaker signals on pachytene chromosomes than on condensed somatic metaphase chromosomes. Therefore, probes with high density of oligos would be recommended for FISH studies on pachytene chromosomes[2].

Potential applications of haplotype-specific FISH probes developed from maize inbred B73 and Mo17

A major limit for the haplotype-specific FISH probes developed based on the B73/Mo17 sequences is that these probes may be used only for studies of materials derived from hybrids between B73 and Mo17. We tested FISH experiments on a hybrid between B73 and a different inbred line and were able to distinguish the two copies of chromosome 10. Thus, it is likely that these probes can be used to study materials derived from hybrids between B73 (Mo17) and another inbred that is relatively different from B73 (Mo17), which will significantly expand the usage (value) of these probes.

Supplementary Table 1. Crossovers identified in the 10 IBMRILs based on genotyping data.

IBMRIL[a]	No. Crossovers[b]	Maximum Inter-Crossover Distance[c]	Minimum Inter-Crossover Distance[d]
Mo029	7	64,979,839	149,833
Mo157	5	58,984,803	2,763,831
Mo177	3	121,452,489	5,627,556
Mo189	3	68,533,558	5,767,401
Mo270	5	87,072,476	852,338
Mo321	5	81,626,009	4,269,607
Mo326	3	64,642,939	13,056,958
Mo328	3	112,853,139	4,956,302
Mo346	7	58,907,705	1,021,159

[a] IBMRIL identifier.

[b] Number of crossover events. For example, an A (B73) to B (Mo17) transition counts as one crossover.

[c] Largest segment of parental DNA (i.e. maximum distance in basepairs between two crossover events or between the telomere and the first/last crossover event). A crossover event is positionally defined as the position halfway between the two markers defining the crossover event. Distances are calculated from this point.

[d] Smallest segment of parental DNA (i.e. minimum distance in basepairs between two crossover events or between the telomere and the first/last crossover event). A crossover event is positionally defined as the position halfway between the two markers defining the crossover event. Distances are calculated from this point.

Supplementary References

1 Han, Y., Zhang, T., Thammapichai, P., Weng, Y., & Jiang, J. Chromosome-specific painting in cucumis species using bulked oligonucleotides. *Genetics* **200**, 771-779 (2015).

2 Jiang, J. Fluorescence in situ hybridization in plants: recent developments and future applications. *Chromosome Res.* **27**, 153-165 (2019).
