# Supplementary material for: Meiotic crossovers characterized by haplotype-specific chromosome painting in maize
Source: Nat Commun. 2019 Oct 10;10:4604. doi: 10.1038/s41467-019-12646-z (PMC6787048; doi:10.1038/s41467-019-12646-z)
Supplement: Supplementary file 4 — Description of Additional Supplementary Files [file 41467_2019_12646_MOESM4_ESM.docx]

**Description of Additional Supplementary Files**

File name: Supplementary Data 1

Description: Number of oligos of hapB and hapM probes within each 2 Mb-window.

File name: Supplementary Data 2

Description: Positions of crossovers identified on chromosome 10 in 58 F_2_ plants.

File name: Supplementary Data 3

Description: Crossovers and genotyping data of the 10 IBMRILs used for chromosome painting analysis.

File name: Supplementary Data 4

Description: Sequences of all oligos included in FISH probes hapB and hapM.
