# Supplementary material for: Meiotic crossovers characterized by haplotype-specific chromosome painting in maize
Source: Nat Commun. 2019 Oct 10;10:4604. doi: 10.1038/s41467-019-12646-z (PMC6787048; doi:10.1038/s41467-019-12646-z)
Supplement: Supplementary file 2 — Peer Review [file 41467_2019_12646_MOESM2_ESM.pdf]

Reviewers' comments:

Reviewer #1 (Remarks to the Author):

The manuscript by Martins et al. presents development of a chromosome painting technique that can cytologically distinguish two haplotypes of maize. The authors then use this method to analyze meiotic recombination patterns in a hybrid between the B73 and Mo17 inbred lines, as well as in an intermated B73xMo17 recombinant inbred line population.

The authors provide a very good description of the tool development. Figure 1 clearly demonstrates the specificity of the different oligo pools and allows the reader to understand the composition of the final oligo pools (hapB and hapM). However, the manuscript part focusing on recombination is much less developed. Although the data presented in this manuscript are intriguing, the framework drawn by the authors emphasizes the technique, rather than the biological relevance of the data. I would recommend refocusing the discussion and conclusions on the former. This work can be very interesting when presented properly.

Major notes:
1. A major novelty and advantage of using chromosome painting to study recombination is that this technique can distinguish between the two parental chromosomes in the progeny, which is not possible using sequencing. Additionally, particular chromosomes can be tracked across generations. This advantage can address plethora of fascinating questions related to recombination, yet the authors do not mention this aspect of the work at all.

2. The authors' data from the intermated recombinant inbred lines indicate "accumulation" of COs in pericentromeric regions after only four generations, even though such COs are not observed in the hybrid analysis. This could indicate that the pericentromeric COs are beneficial in some way, but the authors do not discuss this in depth.

3. The issue of chromosomes lacking COs should be explained better. In meiotic recombination, only two of the four chromatids are involved in the CO event. Hence, only one-half of progeny chromosomes will carry evidence of the CO. However, this notion is absent from the discussion, leaving the readers to conclude that there might be bivalents lacking COs altogether.

4. In the first and second sentence of the third paragraph of the discussion, the authors mention that meiotic COs can be estimated using molecular marker and population studies, revealing a variation of 11 to 65 COs between maize inbred lines, citing four papers. However, it's not clear what these numbers represent. Are these COs per meiosis? Furthermore, technically speaking, only one of the papers documents CO rates in inbred lines, which is done using a cytological approach rather than using molecular markers.

Minor notes:
1. The authors discuss numerous times a technique using recombination modules to characterize recombination. It may be worth mentioning that this methopd in maize has only been successfully used in a single inbred line.

2. At the beginning of the second paragraph of the introduction, the authors state that in Arabidopsis, COs are "nearly evenly distributed" along chromosomes. This is vague and disguises the fact that CO hotspots are still present in Arabidopsis. Perhaps a better phrasing would be that "COs are present in all genome regions."

3. In the last sentence of the 'Meiotic COs revealed by haplotype-specific chromosome painting' section, the authors mention that they only found a double CO on one chromosomes out of 116 analyzed and this indicates the presence of CO interference on "this chromosome." Rather, the

authors should clarify that CO interference exists broadly, not just on this one chromosome, or not mention interference specifically at all.

4. Numerous times throughout the manuscript, the authors say, “distal end.” This is redundant. It would be better to say “distal region,” or “chromosome ends.”

Reviewer #2 (Remarks to the Author):

Martins et al. visualized the Cos on maize chromosome 10 using fluorescence in situ hybridization (FISH) by parent-specific oligos. It is a nice technology to characterize CO cytologically. However, this technique relies heavily on the design of probes and genomic information, which limits its use. The whole study is only a technical report, but the lack of biological problems is another clear shortcoming. The authors painted chromosome 10 from 58 F2 and 10 RIL plants, but not explain the purpose and no biological discoveries at all. FISH method must lose a part of COs, because it shows insufficient resolution in peri-telomere, where COs frequency is much higher than other genomic regions. FISH can be completely replaced with cytological observation of late recombination nodules (RNs) or marker proteins, like MLH1. In the other hand, the authors determined CO position based on the longitudinal chromosome axis length during metaphase, but we do not know what it is the biological means, and whether it is of value to other studies. It should be better to observe pachytene bivalents from pollen mother cells, or chromatids from microspores to enable parallel detection of COs from hundreds of gametophytes, but not metaphase cells from F2 and RIL plants.

Minor suggestions:

1. Introduction: It is a technical article, but it lacks a necessary overview of the detection technology itself.
2. “RN-based genome-wide mapping of COs has been reported in tomato and maize.” High resolution CO pattern across 10 chromosomes can be profiled through cytological observation of RNs or CO markers. While FISH can only focus on limited chromosomes, like chromosome 10 of maize in this study with low resolution. So, why not choose to observe RNs or CO markers?
3. “We measured the distance (μm) from each exchange position (EP) to the telomere of the respective chromosome arm.” What does this distance represent? Does it indicate physical position? How to confirm it?
4. “Most EPs were located within intervals 20-40 on the short arm and intervals 10-55 on the long arm...”: “We divided the short and long arms of chromosome 10 into 20 intervals...”, totally 20 intervals, what “intervals 20-40” and “intervals 10-55” represents?
5. Fig.4: Please use simple representation of x-axis, like “1-20”.
6. “...COs were observed in low percentages of cells from the remaining four F2 plants...” What does it mean?
7. “These COs may not be detected by oligo-FISH due to the lack of a sufficient number of oligos selected from the distal ends...”: Fig.2 shows within 6 plants which authors do not observed COs based on FISH, 4 plants actually have CO in peri-telomere, indicates the false negative rate is very high. Therefore, the reviewer does not think the CO set from the 58 F2 plants is reliable.
8. “B73-derived chromosome 10 does not contain a CO. Interestingly, the Mo17-derived chromosome 10 contained putative double COs at 3.86 Mb and 5.42 Mb on the short arm, which resulted in a 1.56-Mb B73 region...”: How to confirm it is a double CO, but not two single COs?
9. “Chromosome preparation from root tips followed published protocols.” and “Amplification and labeling of the oligo probes were according to published protocols.”: Please detailed illustrate the experiment process.

Reviewer #3 (Remarks to the Author):

The MS is extremely well written and easy to read. And I ahve little to 'complain'.
However, the paper is very technical reporting an FISH method to distinguish haplo-chromosomes in maize. The authors demonstrate the techniques using chromosome 10 of maize of inbreds B73 and Mo17. The abstract promises that 'Potential applications of haplotype-specific chromosome painting are discussed'. However I found the discussion rather short, and lacking a marked conclusion section; the authors admit that the probes are applicable only to a specific variety (genotype), but have unique applications in tracking specific chromosomes derived from a single genotype, potentially to analysis of somatic recombination or in polyploids.

Despite, the impressive results, the reported technique is expensive, and cannot be transferred easily to other crops. For each new species, large sequencing and mapping data are needed as well as extensive computational power to design the probes. Additionally, the oligo pools that need synthesising for use as probes will cost several thousand dollars each. For maize, the technique works well and is stunning.

I would like to congratulate the authors for the excellent FISH and figures showing chromosomes with minimal image processing. Residual cross-hybridization is seen in some figures and this is vital to judge the limit of the technique. Overall, the authors are very honest with the cross hybridization they describe. And as long as a significant difference between the two homologous chromosomes is visible, the technique will work well, in particular if the green and red signals are overlaid, and as the authors say 'produced the best contrast' of haplotype-specific FISH signals. Nevertheless, I object to saying that any probe 'did not generate cross hybridization' (see page 4 bottom): it might not be visible, but might still be there. So at least say 'not visible cross hybridization'.

The authors, after proving the technique analyse cross over frequencies and distribution in F2 progeny of the inbred maize B73 and Mo17. The results that cross-overs are reduced at the centromeres and that not more than 3 cross-overs can be seen cytologically, is not novel, but shown here with a unique and unequivocal method. The fact that in some intermating COs might be increased near the centromere has important prospects for breeding.
The authors also describe that a large number of chromosome 10 in the F2 BM plants do not show a CO, and suggest that the two inbred liens are too polymorphic for proper COs to be established. But does this also have an effect on the distribution of COs even if similar to what is expected with lack near the centromeres? And what happens to these chromosome 10s – did the authors see any non-disjunction at meiosis. Do they think that other chromosomes within these plants also have no COs, or is chromosome 10 unique and particularly variable (and hence the technique to distinguish them cytologically by FISH particularly suited). And does the lack of COS have an effect on the fertility of these plants.

Best regards,
Trude Schwarzacher
University of Leicester, UK

**Response to comments from Reviewer #1**:

The manuscript by Martins et al. presents development of a chromosome painting technique that can cytologically distinguish two haplotypes of maize. The authors then use this method to analyze meiotic recombination patterns in a hybrid between the B73 and Mo17 inbred lines, as well as in an intermated B73xMo17 recombinant inbred line population.

The authors provide a very good description of the tool development. Figure 1 clearly demonstrates the specificity of the different oligo pools and allows the reader to understand the composition of the final oligo pools (hapB and hapM). However, the manuscript part focusing on recombination is much less developed. Although the data presented in this manuscript are intriguing, the framework drawn by the authors emphasizes the technique, rather than the biological relevance of the data. I would recommend refocusing the discussion and conclusions on the former. This work can be very interesting when presented properly.

**Response**: We very much appreciate these comments from the reviewer. We have completely re-structured and re-written the Discussion by focusing on the biological relevance of our dataset, including the suppression of recombination in the pericentromeric regions as well as the frequency of chromosome 10 lacking COs in the $F_2$ population.

Major notes:

1. A major novelty and advantage of using chromosome painting to study recombination is that this technique can distinguish between the two parental chromosomes in the progeny, which is not possible using sequencing. Additionally, particular chromosomes can be tracked across generations. This advantage can address plethora of fascinating questions related to recombination, yet the authors do not mention this aspect of the work at all.

**Response**: We have added discussion for the comparison of haplotype-specific FISH with traditional methods, which are based on mapping late recombination nodules (RNs) or CO-associated protein markers on synaptonemal complexes (SCs), for detecting meiotic COs. The RN- or CO marker-based techniques predict the positions of "future" COs that may occur on the SCs. By contrast, FISH reveals the positions of "past or historical" COs resulting from sister chromatid exchange of homologous chromosomes. We were able to characterize multiple COs associated with chromosome 10 in the recombinant inbred lines from the intermated B73 x Mo17 population, which cannot be analyzed by any traditional method. These key pointed are added in the revised manuscript.

2. The authors' data from the intermated recombinant inbred lines indicate "accumulation" of COs in pericentromeric regions after only four generations, even though such COs are not observed in the hybrid analysis. This could indicate that the pericentromeric COs are beneficial in some way, but the authors do not discuss this in depth.

**Response**: We appreciate these comments and have added relevant discussion on COs in pericentromeric regions. Since there was no selection in the development of the intermated recombinant inbred lines, we are not able to predict if these pericentromeric COs are beneficial. But recovery of such CO events in the population suggest that it will be possible to create favorable allelic combinations of genes residing in the recombination-suppressed pericentromeric regions.

3. The issue of chromosomes lacking COs should be explained better. In meiotic recombination, only two of the four chromatids are involved in the CO event. Hence, only one-half of progeny chromosomes will carry evidence of the CO. However, this notion is absent from the discussion, leaving the readers to conclude that there might be bivalents lacking COs altogether.

**Response**: We have added the discussion suggested by the reviewer.

4. In the first and second sentence of the third paragraph of the discussion, the authors mention that meiotic COs can be estimated using molecular marker and population studies, revealing a variation of 11 to 65 COs between maize inbred lines, citing four papers. However, it's not clear what these numbers represent. Are these COs per meiosis? Furthermore, technically speaking, only one of the papers documents CO rates in inbred lines, which is done using a cytological approach rather than using molecular markers.

**Response**: We are sorry for the incomplete sentence. We have rewritten this portion of the Discussion and have cited more relevant papers published in both maize and Arabidopsis. Our analysis showed that the frequency of maize chromosome 10 lacking a CO in the $F_2$ population is within the range compared to similar frequencies of chromosomes in both maize and Arabidopsis in the literature.

Minor notes:

1. The authors discuss numerous times a technique using recombination modules to characterize recombination. It may be worth mentioning that this method in maize has only been successfully used in a single inbred line.

**Response**: We have added the suggested text in the revised manuscript.

2. At the beginning of the second paragraph of the introduction, the authors state that in Arabidopsis, COs are "nearly evenly distributed" along chromosomes. This is vague and disguises the fact that CO hotspots are still present in Arabidopsis. Perhaps a better phrasing would be that "COs are present in all genome regions."

**Response**: We agree with the reviewer's comment. To avoid any possible confusion, we have deleted this sentence and have focused on literature related to "noneven CO distribution along chromosomes" observed in other plant species.

3. In the last sentence of the ‘Meiotic COs revealed by haplotype-specific chromosome painting’ section, the authors mention that they only found a double CO on one chromosomes out of 116 analyzed and this indicates the presence of CO interference on “this chromosome.” Rather, the authors should clarify that CO interference exists broadly, not just on this one chromosome, or not mention interference specifically at all.

**Response**: We have deleted this sentence in the revised manuscript.

4. Numerous times throughout the manuscript, the authors say, “distal end.” This is redundant. It would be better to say “distal region,” or “chromosome ends.”

**Response**: We have replaced “distal end” with “distal regions” in the revised manuscript.

**Response to comments from Reviewer #2**:

Martins et al. visualized the Cos on maize chromosome 10 using fluorescence in situ hybridization (FISH) by parent-specific oligos. It is a nice technology to characterize CO cytologically. However, this technique relies heavily on the design of probes and genomic information, which limits its use. The whole study is only a technical report, but the lack of biological problems is another clear shortcoming. The authors painted chromosome 10 from 58 F2 and 10 RIL plants, but not explain the purpose and no biological discoveries at all. FISH method must lose a part of COs, because it shows insufficient resolution in peri-telomere, where COs frequency is much higher than other genomic regions. FISH can be completely replaced with cytological observation of late recombination nodules (RNs) or marker proteins, like MLH1. In the other hand, the authors determined CO position based on the longitudinal chromosome axis length during metaphase, but we do not know what it is the biological means, and whether it is of value to other studies. It should be better to observe pachytene bivalents from pollen mother cells, or chromatids from microspores to enable parallel detection of COs from hundreds of gametophytes, but not metaphase cells from F2 and RIL plants.

**Response**: We respect and appreciate these comments. Following the reviewer’s comments and concerns on relevant biological significance of our work, we have re-organized and re-written the Introduction and Discussion of our manuscript to better highlight the biological relevance of our technique. Meiotic COs derived from homologous plant chromosomes can be mapped based on various cytological and population-based methods, but they have never been visualized directly on metaphase chromosomes. In the revised manuscript we try to explain the purpose of our study and to highlight the biological relevance of our data derived from haplotype-specific FISH.

We agree with the reviewer about the value of using RNs or marker proteins such as MLH1 for mapping COs. However, RNs or MLH1 are used to predict or map the positions of "future COs" on the synaptonemal complexes (SCs), but cannot be used to map the "past or historical COs" associated with the $F_2$ plants or the recombinant inbred lines from the intermated B73 x Mo17 population.

The haplotype-specific FISH probes do not contain the optimal number oligos, which was restricted by the amount of PAV and SNP variation between B73 and Mo17 sequences. These probes do not generate unambiguous FISH signals on the paired pachytene bivalents. Thus, the hapB and hapM probes can unfortunately only be used to study somatic metaphase chromosomes.

Minor suggestions:

1. Introduction: It is a technical article, but it lacks a necessary overview of the detection technology itself.

**Response**: We have added an overview of traditional CO detection techniques developed for plants in the Introduction.

2. "RN-based genome-wide mapping of COs has been reported in tomato and maize." High resolution CO pattern across 10 chromosomes can be profiled through cytological observation of RNs or CO markers. While FISH can only focus on limited chromosomes, like chromosome 10 of maize in this study with low resolution. So, why not choose to observe RNs or CO markers?

**Response**: We respect these comments from the reviewer. However, each technique has its own strength and weakness for mapping meiotic COs. The RN- or CO marker-based techniques predict the positions of "future" COs that may occur on the synaptonemal complexes (SCs). Haplotype-specific FISH reveals the positions of "historical" COs resulting from sister chromatid exchange of homologous chromosomes. Thus, the RN- or CO marker-based techniques can only be used to map COs in specific maize genotypes, but not the CO products derived from hybrids or intermated population. Most importantly, these traditional techniques cannot be used to detect the multiple COs on chromosome 10 in the recombinant inbred lines from the intermated B73 x Mo17 population. In addition, the synaptonemal complex-based methodologies are highly technical. Estensive training would be required to use these techniques. To the best of our knowledge, cytological mapping of RNs on SCs was mastered by very few plant labs, including the de Jong lab in Wageningen University and Stack/Anderson labs in Colorado State University. All three principal investigators have retired in recent years, which would further limit the training of RN/SC based technique in the future. By contrast, FISH techniques are more routine and have been used by a large number of plant labs. FISH techniques based oligo probes are essentially the same as the traditional FISH technique, except for the development of the probes.

3. “We measured the distance (μm) from each exchange position (EP) to the telomere of the respective chromosome arm.” What does this distance represent? Does it indicate physical position? How to confirm it?

**Response**: This distance, or length (in μm), is used to compare (divide) the length of the respective chromosome arm. This number represents the position of sister chromatid exchange breakpoint on chromosome 10. For example, if the distance from an EP to the telomere of the long chromosome arm is 1 μm, and the length of the long arm is 2.5 μm, then the breakpoint is mapped at a chromosomal position that accounts 40% (1/2.5) of the arm. We have slightly modified the text in both Results and Methods to avoid confusion.

4. “Most EPs were located within intervals 20-40 on the short arm and intervals 10-55 on the long arm...”: “We divided the short and long arms of chromosome 10 into 20 intervals...”, totally 20 intervals, what “intervals 20-40” and “intervals 10-55” represents?

**Response**: We are sorry for our confusing description. We have modified the text by using “100 intervals for each arm”.

5. Fig.4: Please use simple representation of x-axis, like “1-20”.

**Response**: We have modified the x-axis following the reviewer’s suggestion.

6. “...COs were observed in low percentages of cells from the remaining four F2 plants...” What does it mean?

**Response**: We are sorry for the poor description and have modified the text in the revised manuscript.

7. “These COs may not be detected by oligo-FISH due to the lack of a sufficient number of oligos selected from the distal ends...”: Fig.2 shows within 6 plants which authors do not observed COs based on FISH, 4 plants actually have CO in peri-telomere, indicates the false negative rate is very high. Therefore, the reviewer does not think the CO set from the 58 F2 plants is reliable.

**Response**: The reviewer is right, the haplotype-specific FISH technique has a non-negligible false negative rate for detecting meiotic COs. To address this, we estimated the false negative rate by whole-genome sequencing of 10 $F_2$ BM plants (Figure 5). A total of 13 copies of chromosome 10 of these 10 $F_2$ plants were not recombinant chromosomes based on the FISH results. However, we detected recombination in 4 of these 13 chromosomes. Therefore, the false negative rate of the FISH technique is 31% (4/13). The false negative is largely caused by the fact that the FISH probes contained ~600 oligos within the last 5 Mb region on both arms, which are not sufficient to generate consistently detectable signals.

We agree with the reviewer that we are likely underestimating the number of COs. Nevertheless, our manuscript does not focus on identification of an accurate number of COs in the $F_2$ plants, which could be done by sequencing. Our focus is the development of the haplotype-specific FISH technique and its application to visualize COs, especially the discovery and validation of the lack of COs in pericentromeric regions.

8. "B73-derived chromosome 10 does not contain a CO. Interestingly, the Mo17-derived chromosome 10 contained putative double COs at 3.86 Mb and 5.42 Mb on the short arm, which resulted in a 1.56-Mb B73 region...": How to confirm it is a double CO, but not two single COs?

**Response**: We are not sure if we understand this question. Two single COs involved in the same two chromatids would result in a double CO as the one discovered in the present study. But two single COs involved in three or four different chromatids would not have resulted in a B73 fragment inserted in a Mo17 chromosome 10 (Figure 5, BM48 plant).

9. "Chromosome preparation from root tips followed published protocols." and "Amplification and labeling of the oligo probes were according to published protocols.": Please detailed illustrate the experiment process.

**Response**: The only novel part of the haplotype-specific FISH technique is the identification of PAV- and SNP-based oligos for probe development. Chromosome preparation and labeling of oligo-based FISH probes are the same as our regular oligo-FISH techniques. These procedures are very long and were described in detail previously (Han et al. 2015 for probe labeling; Braz et al. 2018 for chromosome preparation).

**Response to comments from Reviewer #3** (Prof. Trude Schwarzacher):

The MS is extremely well written and easy to read. And I have little to 'complain'.
However, the paper is very technical reporting an FISH method to distinguish haplo-chromosomes in maize. The authors demonstrate the techniques using chromosome 10 of maize of inbreds B73 and Mo17. The abstract promises that 'Potential applications of haplotype-specific chromosome painting are discussed'. However I found the discussion rather short, and lacking a marked conclusion section; the authors admit that the probes are applicable only to a specific variety (genotype), but have unique applications in tracking specific chromosomes derived from a single genotype, potentially to analysis of somatic recombination or in polyploids.

**Response**: We appreciate these comments. We have completely re-written the Discussion and have tried to strengthen the discussion on the biology related to meiotic COs. We have deleted the sentence 'Potential applications of haplotype-specific chromosome painting are discussed' in the abstract.

Excitingly, we have already found that our probes based on B73 and Mo17 can be used to distinguish the two copies of chromosomes in other hybrids with only B73 or Mo17 as one of the parental lines. We have already obtained exciting results in chromosome pairing studies using tetraploid maize lines (derived from diploid hybrids). We will soon develop new manuscripts based on applications of the haplotype-specific FISH probes developed in the current project.

Despite, the impressive results, the reported technique is expensive, and cannot be transferred easily to other crops. For each new species, large sequencing and mapping data are needed as well as extensive computational power to design the probes. Additionally, the oligo pools that need synthesising for use as probes will cost several thousand dollars each. For maize, the technique works well and is stunning.

**Response**: Arbor Biosciences currently charges $1500 for 25,000 oligos (45 nt). Probes hapB and hapM contain 14,498 and 13,753 oligos, respectively. Thus, the total cost for these two probes would be less than $3000, and these two probes can be used indefinitely (each synthesized probe can be used for a million application, see Han et al. 2015, Genetics). Although this technique is still expensive for many plant labs, the cost of DNA synthesis is expected to drop in the future. We should also be able to develop chromosome-specific probes in allopolyploid species using a similar probe developing strategy based on PAV and SNP. For example, we should be able to develop painting probes specific to chromosome 1A, 1B, and 1D, respectively, in wheat. In summary, although it is still expensive to develop haplotype-specific FISH probes, such probes provide an exciting new tool that enable various types of chromosomal studies that were previously not possible.

I would like to congratulate the authors for the excellent FISH and figures showing chromosomes with minimal image processing. Residual cross-hybridization is seen in some figures and this is vital to judge the limit of the technique. Overall, the authors are very honest with the cross hybridization they describe. And as long as a significant difference between the two homologous chromosomes is visible, the technique will work well, in particular if the green and red signals are overlaid, and as the authors say ‘produced the best contrast’ of haplotype-specific FISH signals. Nevertheless, I object to saying that any probe ‘did not generate cross hybridization’ (see page 4 bottom): it might not be visible, but might still be there. So at least say ‘not visible cross hybridization’.

**Response**: We agree with the reviewer on these comments. We have modified the sentence as “the B73 PAV probe generated only minimal cross hybridization to chromosome 10 from Mo17, and vice versa”.

The authors, after proving the technique analyse cross over frequencies and distribution in F2 progeny of the inbred maize B73 and Mo17. The results that cross-overs are reduced at the centromeres and that not more than 3 cross-overs can be seen cytologically, is not novel, but shown here with a unique and unequivocal method. The fact that in some intermating COs might

be increased near the centromere has important prospects for breeding.

**Response**: We fully agree with these comments with the reviewer. One of the most important contributions of our study is the validation of the presence of COs in recombination suppressed regions in the intermating population. We have emphasized this in the Discussion in the revised manuscript.

The authors also describe that a large number of chromosome 10 in the F2 BM plants do not show a CO, and suggest that the two inbred liens are too polymorphic for proper COs to be established. But does this also have an effect on the distribution of COs even if similar to what is expected with lack near the centromeres? And what happens to these chromosome 10s – did the authors see any non-disjunction at meiosis. Do they think that other chromosomes within these plants also have no COs, or is chromosome 10 unique and particularly variable (and hence the technique to distinguish them cytologically by FISH particularly suited). And does the lack of COs have an effect on the fertility of these plants.

**Response**: We are in the process of developing haplotype-specific FISH probes for all 10 maize chromosomes. Our analysis showed that the level of sequence divergence of chromosome 10 (between Mo17 and B72) is similar to the remaining nine chromosomes. Thus, chromosome 10 is not unique and particularly variable. In addition, the Mo17/B73 hybrid was the basis of the hybrid corn industry for years. Thus, any unusual sterility, resulting from non-disjunction, would have to be noticed.

Our FISH data indicated that 60 of 116 copies of chromosome 10 from the 58 $F_2$ BM plants are not recombinant chromosomes. Our sequencing data (Figure 5, also see our response to comments to reviewer #2) showed that 69% (9/13) of such FISH-negative chromosome 10 were confirmed to be non-recombinant chromosomes. Therefore, we estimate that the percentage of chromosome 10 without CO is 36% (69% x 60/116) in the $F_2$ population. In comparison, approximately 30% (423 of 1404) chromosome 4 of Arabidopsis in a Columbia x Landsberg $F_2$ population do not contain CO. We have added this analysis in the Discussion in the revised manuscript.

Reviewers' comments:

Reviewer #1 (Remarks to the Author):

The authors have dealt very well with revisions requested by myself as well as reviewer 3. I believe the revised manuscript will be of interest to a wide community of scientists. I have, however, two minor comments for consideration for the authors and editor. First, the second paragraph of Discussion will fit much better in the Results section. Second, authors' comments on the cost of producing labelled oligos as well as limitations of the method in terms of labeling density made in response to Reviewer 2 may be actually quite useful for some readers. I would suggest including them as supplementary material.

Reviewer #2 (Remarks to the Author):

The authors have a detail response for the raised questions. I agree with the significance of the method for visualizing COs, however, I still have some concerns for the high false positive rate as I and authors agreed in Q7 and A7 which may limit the application to "discovery and validation of the lack of COs in pericentromeric regions" as authors proposed in A7. Another concern is the phenomenon (lack of COs in pericentromeric regions) had been found for decades in many species including maize, what is underlying biological problem to be answered by using this method?

Additional concern in Q8: What this reviewer confused is how to distinguish double CO occurred in same chromatids and two single COs in different chromatids. As Fig.5 BM48 showed, there are two conditions: 1) two COs are located in 3.86Mb and 5.42Mb along M2 chromosome, respectively; 2) one CO is located in 3.86Mb along B1 chromosome and another is in 5.42Mb along M2 chromosome. Due to the weak signals in peri-telomere, the reviewer thinks these two conditions cannot be distinguished, therefore complicating further analysis.

**Response to comments from Reviewer #1**:

The authors have dealt very well with revisions requested by myself as well as reviewer 3. I believe the revised manuscript will be of interest to a wide community of scientists. I have, however, two minor comments for consideration for the authors and editor. First, the second paragraph of Discussion will fit much better in the Results section.

**Response**: Following the Reviewer's suggestion we have deleted some of the text of this paragraph and merged the remaining text with the last section in Results.

Second, authors' comments on the cost of producing labelled oligos as well as limitations of the method in terms of labeling density made in response to Reviewer 2 may be actually quite useful for some readers. I would suggest including them as supplementary material.

**Response**: Following the Reviewers' suggestion we have developed a "Supplemental Research Note" to include the information. This Supplemental Research Note is indicated in both Results and Methods, thus, interested readers should be able to readily find the Note. Information covered by this Note includes: (1) Cost of oligo-based FISH probes; (2) Oligo-FISH probes for somatic metaphase chromosomes or meiotic pachytene chromosomes; and (3) Potential applications of haplotype-specific FISH probes developed from maize inbred B73 and Mo17.

**Response to comments from Reviewer #2**:

The authors have a detail response for the raised questions. I agree with the significance of the method for visualizing COs, however, I still have some concerns for the high false positive rate as I and authors agreed in Q7 and A7 which may limit the application to "discovery and validation of the lack of COs in pericentromeric regions" as authors proposed in A7.

**Response**: Our FISH technique has a "high false negative rate", but not a "high false positive rate", for detecting meiotic COs. We specifically addressed this "false negative rate" in our last response. The false negative is caused by the fact that our FISH probes contained ~600 oligos within the last 5 Mb regions on both arms, which are not sufficient to consistently generate detectable FISH signals. Thus, COs located at the terminal regions may not be detected by FISH. However, a CO located in the pericentromeric region, which would carry a large recombined chromosome segment, can be readily detected. Thus, the FISH technique is actually ideal for validating COs located in pericentromeric regions.

Another concern is the phenomenon (lack of COs in pericentromeric regions) had been found for decades in many species including maize, what is underlying biological problem to be answered by using this method?

**Response**: We agree with the Reviewer that the phenomenon of lack of COs in pericentromeric regions had been found for decades in many species, including maize. We also agree that our manuscript does not reveal the mechanism underlying this biological phenomenon, which cannot be addressed by the FISH method and is also beyond the scope of our research.

Additional concern in Q8: What this reviewer confused is how to distinguish double CO occurred in same chromatids and two single COs in different chromatids. As Fig.5 BM48 showed, there are two conditions: 1) two COs are located in 3.86Mb and 5.42Mb along M2 chromosome, respectively; 2) one CO is located in 3.86Mb along B1 chromosome and another is in 5.42Mb along M2 chromosome. Due to the weak signals in peri-telomere, the reviewer thinks these two conditions cannot be distinguished, therefore complicating further analysis.

**Response**: We are sorry for misunderstanding the question in our last response. We agree with the Reviewer that we cannot exclude the second possibility (two COs located on B1 and M2, respectively) based on our FISH results. We did observe weak hapB FISH signals on the Mo17-derived chromosome 10, and vice versa, but only in 35% (9/26) and 8% (2/26) of the metaphase cells, respectively. Thus, it is possible that two single COS, rather than a double COs, are associated with BM48. We have added the two possibilities to explain BM48 in the revised manuscript. We thank the Reviewer for catching this.

REVIEWERS' COMMENTS:

Reviewer #2 (Remarks to the Author):

the authors have addressed my concerns well and congratulations!
